# Integrated dual-tomography for refractive index analysis of free-floating single living cell with isotropic superresolution

**DOI:** 10.1038/s41598-018-24408-w

**Published:** 2018-04-13

**Authors:** Vinoth B., Xin-Ji Lai, Yu-Chih Lin, Han-Yen Tu, Chau-Jern Cheng

**Affiliations:** 1 Institute of Electro-Optical Science and Technology, National Taiwan Normal University, Taipei, 11677 Taiwan; 2 Department of Electrical Engineering, Chinese Culture University, Taipei, 11114 Taiwan

## Abstract

Digital holographic microtomography is a promising technique for three-dimensional (3D) measurement of the refractive index (RI) profiles of biological specimens. Measurement of the RI distribution of a free-floating single living cell with an isotropic superresolution had not previously been accomplished. To the best of our knowledge, this is the first study focusing on the development of an integrated dual-tomographic (IDT) imaging system for RI measurement of an unlabelled free-floating single living cell with an isotropic superresolution by combining the spatial frequencies of full-angle specimen rotation with those of beam rotation. A novel ‘UFO’ (unidentified flying object) like shaped coherent transfer function is obtained. The IDT imaging system does not require any complex image-processing algorithm for 3D reconstruction. The working principle was successfully demonstrated and a 3D RI profile of a single living cell, Candida rugosa, was obtained with an isotropic superresolution. This technology is expected to set a benchmark for free-floating single live sample measurements without labeling or any special sample preparations for the experiments.

## Introduction

Cell RI is a key parameter to determine or correlate with biophysical parameters and cell metabolic activities, these are related with dry mass, wet mass, protein concentration, elasticity, cell division, infection, etc. Studying RI value in a bulk population can yield only an average data whereas single cell study gives more quantitative RI distribution for the analysis at sub-cellular level^1–3^. Recently, diffraction phase tomography^4–13^ has been applied to obtain RI distributions either through beam rotation or sample rotation. Digital holographic microtomography (DHµT) is one of the promising imaging technique developed for mapping the refractive index (RI) profiles of biological specimens in three dimensions (3D)^4–10^. The spatial frequencies imposed by the finite numerical aperture (NA) of the objective lens are collected during image reconstruction by combining the frequency bands corresponding to different angles that is, the synthetic aperture method^11,12^, illumination rotation method^14^, one can improve the lateral resolution of imaging^10–18^. In beam rotation tomography, the aforementioned spatial frequency collection constraint is commonly referred to as the missing cone problem^17^; it results in a low axial resolution. In the sample rotation tomography method, the axial resolution can be improved by rotating the sample^19–24^, the sample is loaded into microcapillary or micropipette and rotated mechanically^4,24–27^. Consequently, achieving a full ${360}^{{\rm{o}}}$ rotation of the specimen is difficult, and mechanical rotation makes the system susceptible to aberrations^23–25^. The limited rotation angle of the specimen results in missing apple core (MAC) data information along the optical axis^17,23^. Recently, optical tweezers^28–32^, microfluidic channel^33^ and flow cytometer^34^ techniques have been developed for sample rotation; using digital holography technique to record the object wavefronts without any unwanted mechanical perturbations in the system^28–34^ in addition to allowing a full-angle rotation of the sample^32,33^. Thus, the so-called MAC problem can be overcome^32^, resulting in full freedom to control the sample without any external perturbation and any special sample preparations for the experiments. The obtained MAC data can be combined with the beam rotation results to extend the spatial frequency coverage. However, till date, an integrated system capable of performing a full angle specimen rotation along with beam rotation has not been developed, especially for free-floating samples; our study developed such a system for the first time. And the important thing is, most of the biological cells are live only in the free-floating condition^1–3^. Such a dedicated system will revolutionize the biological and pharmaceutical fields where a non-invasive single cell study is necessary for drug delivery and interaction studies^35^. In addition, measuring RI distribution of cell in a free-floating condition is extremely important because the RI profile is highly dependent on the cell condition such as chemical treatments, live or dead, *etc*^1–3^.

## Methodology

**Figure 1:**
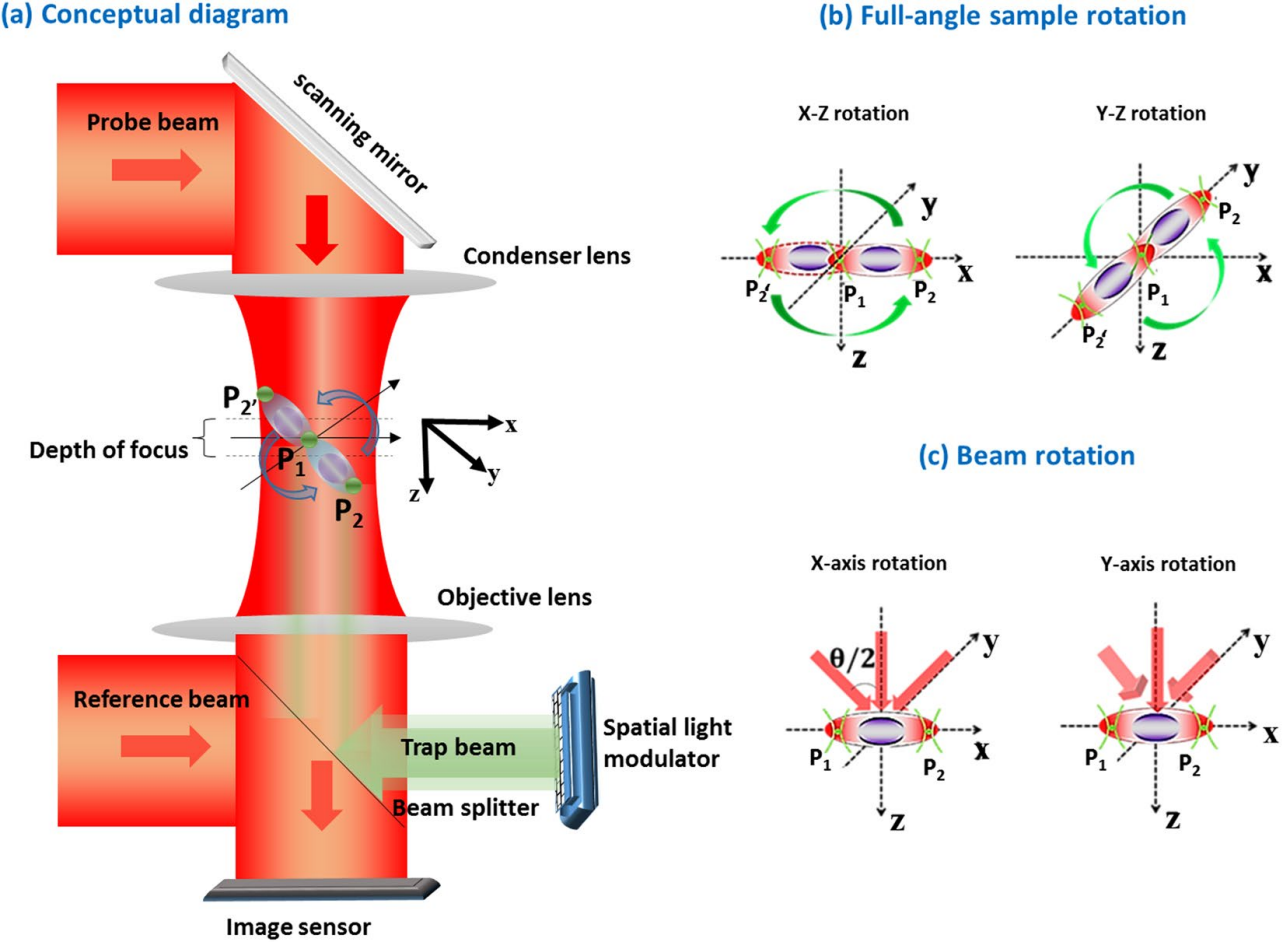
(**a**) Conceptual representation of the IDT imaging system; (**b**) full-angle rotation scheme (P_1_: fixed trap and P_2_: rotation trap), P_1_ and P_2_ are initial traps and P_2_′ shows the trap rotation at different instances; (**c**) beam rotation along the x and y axis respectively.

To the best of our knowledge, we are the first to develop an integrated dual-tomographic (IDT) imaging system for combining the spatial frequencies of full-angle specimen rotation with those of beam rotation for free-floating single live cell RI map generation. The conceptual diagram of the proposed IDT imaging system is shown in Fig. 1. The red line in Fig. 1(a) represents the imaging system and the green line represents the holographic optical tweezers system. In the imaging system, the probe beam carries sample information while passing and creates an interference with a reference beam, which is a collimated Gaussian beam, and the holograms are recorded by an image sensor. P_1_ and P_2_ are the two trap points generated from a computer-generated hologram (CGH) displayed using a spatial light modulator (SLM). The Gerchberg–Saxton (GS) algorithm^36^ with Fresnel propagation is applied to synthesize a series of CGHs and is displayed in the SLM to create trap points P_1_ and P_2_ respectively, refer Supplementary document for more details about GS algorithm used.

**Figure 2:**
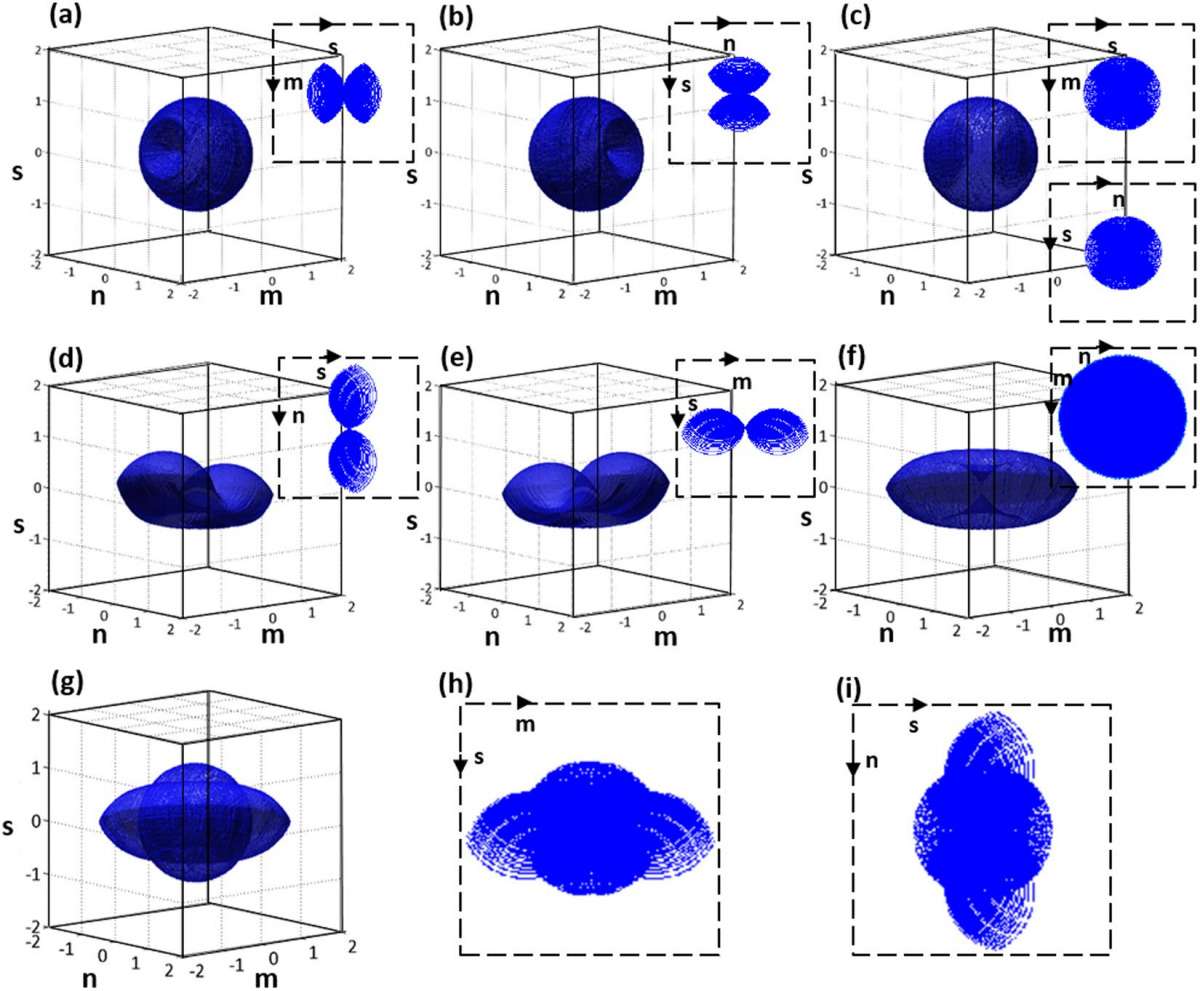
Coherent transfer functions (CTFs), (**a**,**b**) shows the sample rotation CTFs along the x-z & y-z directions, (**c**) full-angle sample rotation CTF: (**a**) and (**b**) are combined to obtain a spherical CTF; (**d**,**e**) beam rotation along the x- and y -axis; (**f**) beam rotation along the x-y direction offers extended lateral frequency coverage but limited axial resolution; (**g**) CTF of IDT imaging method: combined (**c**,**f**) results in a ‘UFO’ (unidentified flying object) like shaped CTF; (**h**,**i**) sectional views along m-s and n-s directions, which shows the extended axial frequency coverage.

The full-angle sample rotation scheme is shown in Fig. 1(b). Here, P_1_ is a fixed trap that holds the sample and P_2_ trap is used to rotate the sample along the x-z and y-z directions, as shown in Fig. 1(b). For more details about the rotation procedures, please ref.^32^. After full-angle data collection, the sample is trapped at a fixed point. The probe beam is steered using a two-dimensional (2D) scanning mirror pivoted along the x- and y-axis to obtain the object frequencies at different illumination angles, as shown in Fig. 1(c). The corresponding holograms are captured by the image sensor for reconstruction. Then, the collected spatial frequencies of object at different angles are mapped to a Ewald sphere to generate a 3D image of the object^16,19^. The 3D coherent transfer functions (CTF) is constructed and shown in Fig. 2. Here, (m, n, s) are the Fourier frequencies along (x, y, z) directions. The 3D CTF of sample rotation along the x-z direction is shown in Fig. 2(a); the MAC problem occurred along the m-axis. Figure 2(b) shows the3D CTF of sample rotation along the y-z direction and the MAC problem occurs along the n-direction. In both x-z and y-z rotations, the center-sliced CTFs are shown as insets in Fig. 2(a,b) respectively, to visualize the MAC problem as well as the missing isotropic frequency coverage. To overcome the MAC problem and achieve isotropic resolution, the obtained spatial frequencies of x-z and y-z directions are combined^32^. Consequently, a complete spherical spatial frequency coverage is obtained as shown in Fig. 2(c), for 3D CTF and gives an isotropic resolution of (λ⁄2NA)^17,26^.

In Fig. 2(c), the inset figure shows the expanded spatial frequency coverage along the MAC data region, and an isotropic spatial frequency coverage is obtained. The 3D CTFs corresponding to the beam rotation along x- and y-directions are shown in Fig. 2(d,e), respectively. The illumination beam is completely scanned along the x- and y-directions to achieve the expanded lateral spatial frequency coverage along m- and n-directions with the lateral resolution of (λ⁄4NA)^17,26^; consequently, a ‘doughnut’ like shaped CTF is obtained, as shown in Fig. 2(f). Here an enlarged spatial frequency is obtained and its spatial resolution can achieve less than half of the wavelength, such spatial resolution can be referred in different names as, resolution enhancement, super-resolved, sub-diffraction limit or superresolution^11,18,19,37^. And Fig. 2(f) shows a weaker bandwidth coverage along the axial direction compared with the CTF of full-angle sample rotation. One of the main advantages of the proposed IDT imaging system is the ability to combine (Fig. 2(c,f)) the spatial frequencies of the full-angle sample rotation (obtained MAC data) with the beam rotation spatial frequencies. Consequently, ‘UFO’ (unidentified flying object) like shaped CTF is obtained with extended spatial frequency coverage, as shown in Fig. 2(g). The sectional views along the m-s and n-s directions are shown in Fig. 2(h,i), and the spatial frequency extension along the axial direction can be clearly observed. The cutoff frequency distribution along axial $({{\rm{F}}}_{{\rm{z}}}^{{\rm{IDT}}})$ and lateral directions $({{\rm{F}}}_{{\rm{x}},{\rm{y}}}^{{\rm{IDT}}})$ for the IDT system is given by^26^,

1 $${{\rm{F}}}_{{\rm{x}},{\rm{y}}}^{{\rm{IDT}}}=\frac{4{\rm{NA}}}{{\rm{\lambda }}},{{\rm{F}}}_{{\rm{z}}}^{{\rm{IDT}}}=\frac{2{\rm{NA}}}{{\rm{\lambda }}}$$

## Experiments and Results

**Figure 3:**
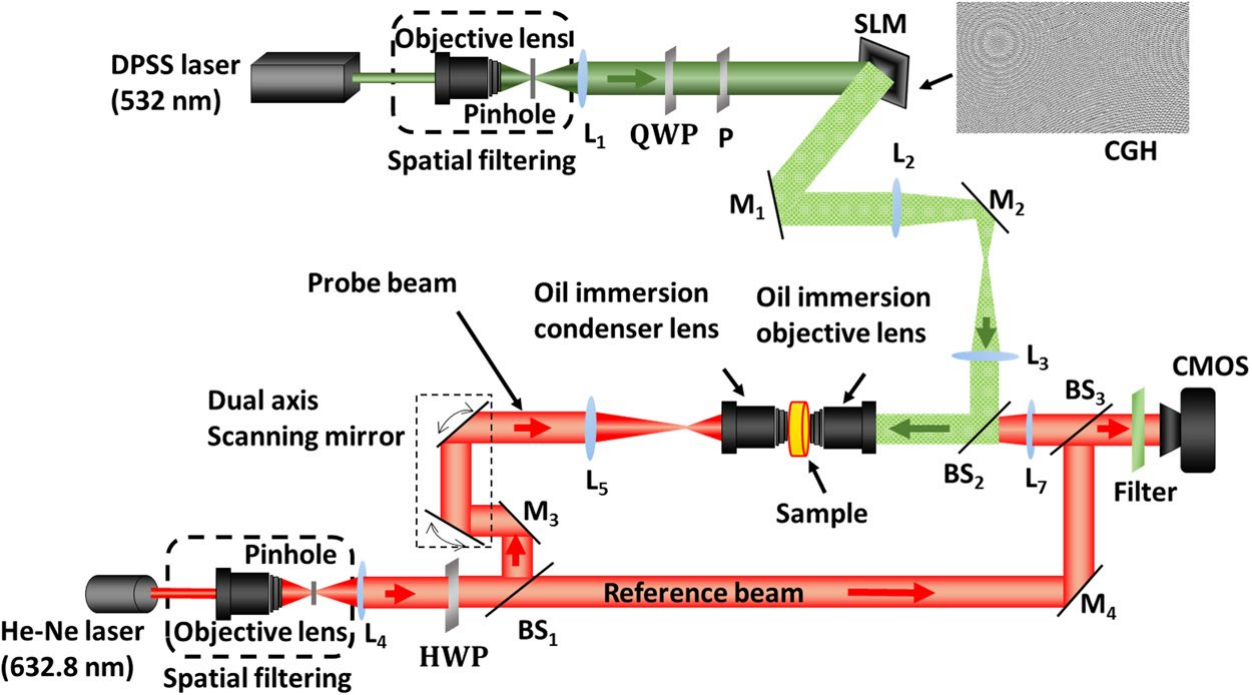
Experimental setup. QWP: quarter wave plate, P: polarizer, HWP: half-wave plate, BS: beam splitter, and CF: color filter is used to stop the green laser interference while recording the holograms.

The experimental implementation of the IDT imaging system comprises holographic optical tweezers with a spatial light modulator (SLM) and a digital holographic microscope (DHM), as shown in Fig. 3. The holographic optical tweezers system (green representation) comprises a diode-pumped solid-state (DPSS) laser source emitting at 532 nm, which is spatially filtered and collimated. The collimated beam is incident on a reflective-phase only SLM, and the reflected beam from the SLM is passed through a 4 F system and imaged by an oil immersion objective at the sample. A series of CGHs is displayed on the SLM to create the twin trap points on the sample for trapping and rotation. The rotation trap can rotate the sample at a full-angle with a step-angle accuracy of 1°; for detailed rotation principle, see ref.^32^. The DHM system (red representation) comprises a modified Mach-Zehnder interferometer configuration; He–Ne laser (632.8 nm) is used as a source, and the beam is spatially filtered and collimated. The collimated probe beam is controlled by a 2D galvo-mirror and combined with the reference beam at an off-axis configuration (for more details about experimental setup refer Supplementary document).

To collect full-angle sample rotation information, the sample is trapped and rotated along the x-z direction to obtain the sample’s spatial frequencies in this direction. Similarly, the sample is rotated along the y-z direction to obtain the corresponding spatial frequencies. The lateral and axial resolution for the full-angle sample rotation case is approximately 320 nm (λ⁄2NA). To collect beam rotation information, the sample is trapped and the illumination beam is rotated along x-and y-directions using the 2D galvo-mirror. The lateral resolution of the beam rotation case is approximated to be 160 nm (λ ⁄4NA). The corresponding holograms are recorded by using a complementary metal-oxide-semiconductor (CMOS) image sensor. For experimental validation, free-floating live yeast—candida rugosa (ATCC 200555) —is used as the sample.

**Figure 4:**
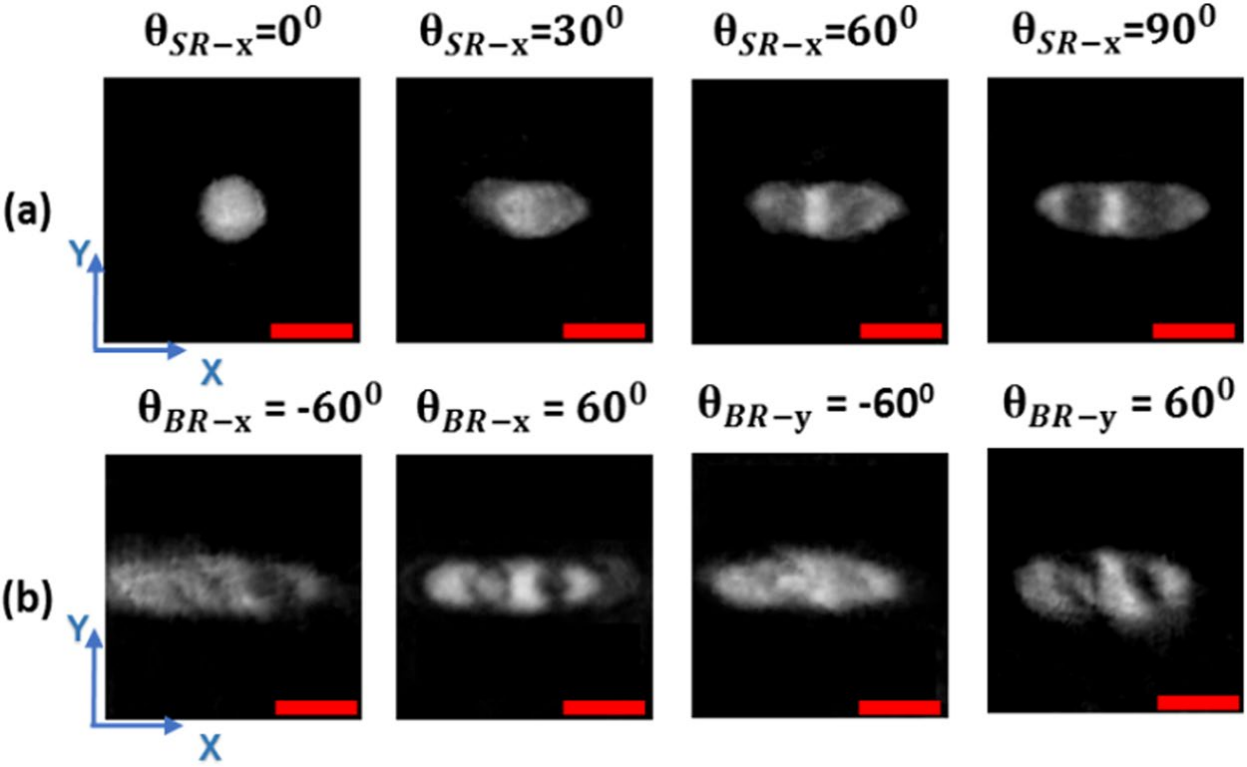
Reconstructed phase results (**a**) x-axis sample rotation results at different rotation angles, (90° result is the same as a normal incident result), (**b**) inclined angle information of beam rotation along x and y-axis respectively. Scale bar: 3 µm. (visualization 1, visualization 2).

**Figure 5:**
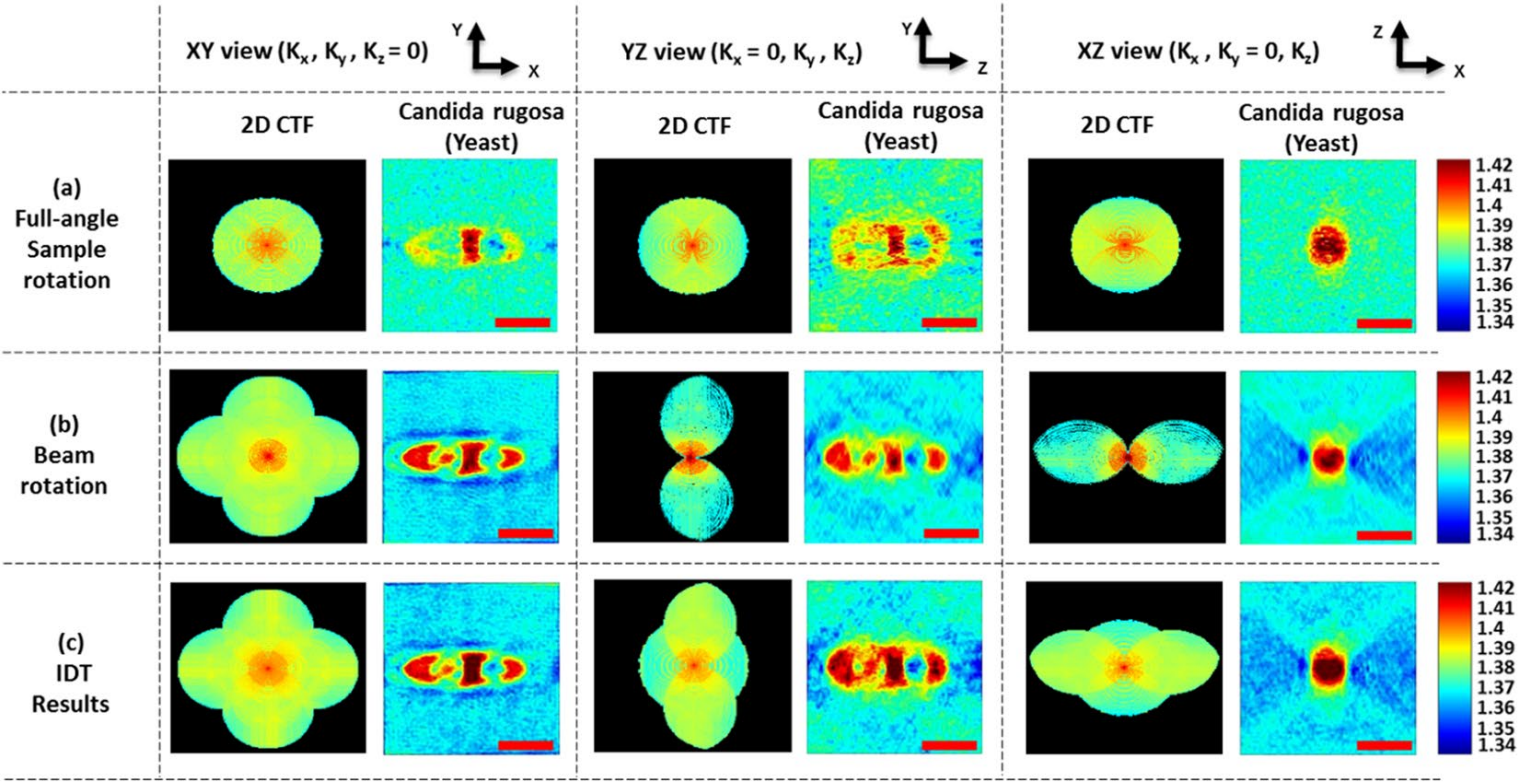
Comparison of tomographic reconstructed results (of live Candida rugosa) at different views with 2D frequency coverage of the object function (**a**) full-angle sample rotation results, (**b**) beam rotation results; (**C**) IDT results (combining (**a**) and (**b**)). Color bar represents the quantitative RI values which vary from 1.34 to 1.42. Scale bar: 3 µm.

First, a single live cell is trapped and isolated from the bulk population, and the yeast is then rotated from 0° to 360° along x-z and y-z directions; for detailed rotation procedures, ref.^32^. The transmitted wavefronts along these directions are recorded by the DHM system. To collect beam rotation information, the Candida rugosa is trapped at a fixed point. Then, the illumination beam is rotated along x- and y-directions using the 2D galvo-mirror, and the maximum scanning angle achieved is ±60°. The holograms are recorded at an angle of every 1°. The total data acquisition time to complete both beam and sample rotation processes is less than 90 secs, and this can be further decreased by careful optimization in the switching process of CGH display and the balance of trapping mechanism^38^ in the free-floating medium. The holograms are reconstructed and numerically focused, and the focused images were aligned to the central position^32^. The reconstructed phase results of the x-z sample rotation at different angles $({\theta }_{{\rm{S}}{\rm{R}}-{\rm{x}}})$ vary from 0° to 90° as shown in Fig. 4(a); they indicate the clearly focused inner structure of the Candida rugosa even at the 30° rotation information, ref.^32^ for the reconstruction process. The high-frequency information at an inclined angle beam rotation corresponds to x − $({\theta }_{{\rm{B}}{\rm{R}}-{\rm{x}}})$ and y-axis $({{\rm{\theta }}}_{{\rm{BR}}-{\rm{y}}})$, as shown in Fig. 4(b). Then, the reconstructed spatial frequencies at different angles are mapped to the frequency spectrum of Ewald’s sphere to reconstruct the 3D tomography image^32^ without using any complex image-processing algorithm. The 3D tomographic image of Candida rugosa is reconstructed, and three different sectional views (x-y, y-z and x-z) of full-angle sample rotation, combined beam rotation and the IDT results are compared in Fig. 5.

**Figure 6:**
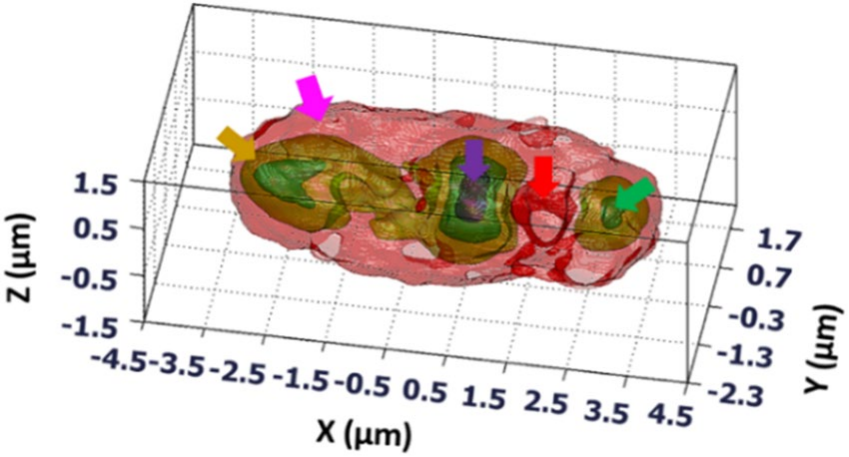
Reconstructed 3D subcellular tomography of Candida rugosa. Yellow arrow: cytoplasm, pink arrow: cell wall, violet arrow: nucleus, red arrow: vacuole, green arrow: mitochondria, (visualization 3, visualization 4).

The full-angle sample rotation results in Fig. 5(a) along the x-y direction indicate the extended axial frequency coverage, and the Candida rugosa outer cell wall information can thus be obtained. In the y-z and x-z directions, the axial information can be observed, which is not clearly visible in the beam rotation results. The corresponding reconstructed 2D CTFs shows the circular spatial frequency coverage for all the three directions, and the MAC data information can thus be obtained. Some interesting things can be observed in the combined beam rotation tomography results. In Fig. 5(b), the internal structures are more clearly visible in the x-y and y-z directions, which can be observed only in the beam rotation tomography results (but not in the sample rotation results) because of high-frequency coverage along the lateral directions. The beam rotation 2D CTFs along different directions indicate the spatial frequency coverage extended along the lateral directions. In full- angle sample rotation, high axial spatial frequency coverage but limited lateral spatial frequency extensions compared with the beam rotation process, which can be clearly observed from the CTFs in Fig. 5(a,b). The most excited IDT tomographic results are shown in Fig. 5(c), in x-y direction, the internal structures of Candida rugosa in the central region can be clearly observed. In y-z direction, the internal structures bonding between the corresponding organelles and a complete structure of the Candida rugosa is obtained, which is not clearly visible in full-angle sample rotation as well as the beam rotation results. In x-z direction, the outer cell wall region information is enhanced which can be obtained from the full-angle sample rotation results. For clear understanding 2D CTFs are shown, in x-y direction, 2D CTF looks the same as that with beam rotation but can be differentiated from the obtained object internal information. In y-z and x-z direction, the ‘UFO’ like shaped CTFs are obtained with extended spatial frequency coverage in the axial direction, which is a novel CTF, which can be clearly observed in the tomographic sectional images. The color bar represents the quantitative RI distribution of live Candida rugosa, and it is varied from 1.34 to 1.42. The standard deviation of the RI distribution corresponding to the surrounding medium is calculated and estimated the RI accuracy of the IDT system is 0.003. Moreover, from the background information, the phase accuracy of the DHM system is approximated as 1 nm (<0.6°). The experimental resolution can be estimated from the boundary response^13,17,19,26,32^ of the reconstructed yeast image, the axial resolution was approximated as 310 nm and the lateral resolution as 150 nm (refer supplementary document for more details). The 3D tomogram of the Candida rugosa is shown in Fig. 6. From the RI distribution of yeast, the contour cell wall structure (pink arrow) and its subcellular structures such as vacuole (red arrow), cytoplasm (yellow arrow), nucleus (violet arrow) and mitochondria (green arrow) can be obtained, as shown in Fig. 6.

## Conclusion

In conclusion, in this study we developed a novel IDT imaging system to combine the spatial frequencies of full-angle sample rotation method along with those of the beam rotation method especially, for free-floating single live cell measurement with isotropic superresolution. The proposed IDT imaging system was successfully demonstrated with free-floating single living cell, Candida rugosa, and its 3D RI distribution was obtained with an accuracy of 0.003. A novel ‘UFO’ like shaped CTF is obtained. The IDT system does not require any complex image-processing algorithm for the 3D reconstruction. We strongly believe that the IDT system has potential applications in the biomedical field where single live cell measurement is much needed for the analysis as well as noninvasive biological studies^1,2^. Moreover, different shaped sample rotation are possible by adapting a suitable trapping procedures^38,39^. Because the IDT system has full control over the free-floating sample, it is possible to extend the spatial frequency coverage along the axial direction. With flexible control of the free-floating sample, the IDT system can also contribute to the paramedical field, where drug–cell chemical interaction study is required^35^.

## Electronic supplementary material

Supplementary document

Visualization-1

Visualization-2

Visualization-3

Visualization-4

Visualization-s1
