# Supplementary material for: Integrated dual-tomography for refractive index analysis of free-floating single living cell with isotropic superresolution
Source: Sci Rep. 2018 Apr 13;8:5943. doi: 10.1038/s41598-018-24408-w (PMC5899089; doi:10.1038/s41598-018-24408-w)
Supplement: Supplementary file 1 — Supplementary document [file 41598_2018_24408_MOESM1_ESM.docx]

**Integrated dual-tomography for refractive index analysis of free-floating single living cell with isotropic superresolution**

B. Vinoth,^1^ Xin-Ji Lai,^1^ Yu-Chih Lin,^1^ Han-Yen Tu,^2^ and Chau-Jern Cheng ^1,^ *

^1^Institute of Electro-Optical Science and Technology, National Taiwan Normal University, Taipei 11677, Taiwan.

^2^Department of Electrical Engineering, Chinese Culture University, Taipei 11114, Taiwan.

*Corresponding author: [cjcheng@ntnu.](mailto:cjcheng@ntnu.)edu.tw

This document provides supplementary information to “Integrated dual-tomography for refractive index analysis of free-floating single living cell with isotropic superresolution”, it describes the Computer-generated hologram (CGH) generation for trapping and rotation spot design based on Gerchberg-Saxton (GS) algorithm which is used to trap and rotate the sample, experimental setup used to implement the integrated dual-tomographic concept, GS algorithm experimental results and the spatial resolution estimation method.

1. Trapping and rotation spot design

The trapping and the rotation spot is generated by the holographic optical tweezers system, which is used to trap and rotate the sample. A series of computer-generated hologram (CGH) is displayed on the spatial light modulator (SLM) to generate the fixed and rotation spot on the sample plane. The experimental setup of the tweezers system is discussed in next section. The CGH is designed based on Gerchberg–Saxton (GS) algorithm^1^. Fig. S1 shows the CGH design flowchart, and the steps used to generate the CGH patterns are as follows:

Step1: The point-source amplitude image with zero phase (ϕ = 0) is designed and used as an input.

Step2: The targeted phase difference matrix $\left( \phi_{n}^{\mathrm{in}} \right)$ is multiplied by the input amplitude wave to generate the complex wavefront information.

Step3: The complex wavefront is multiplied with convex lens at SLM plane and Fresnel propagation is applied to propagate to reconstruction plane to produce the targeted phase information $\left( \phi_{n}^{t} \right)$ at different focal planes.

Step4: The same is back-propagated from the reconstruction plane to SLM plane, and then the concave lens profile is multiplied to obtain its phase change profile back at the SLM plane.


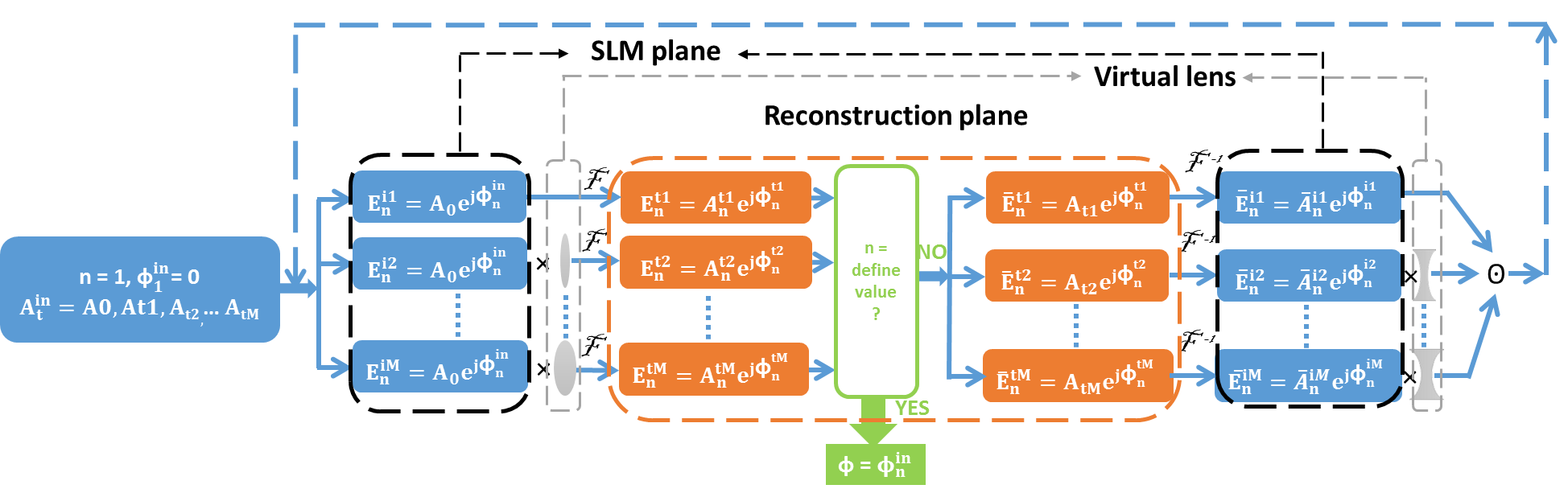


Fig. S1. Flow chart explanation of Gerchberg-Saxton algorithm to generate the CGH which will be used to generate trapping and rotation spots. A_0_: incident wave amplitude, A_t_: target wave amplitude, the symbol ʘ: convolution representation, F -Fourier transform, and F ^-1^- Inverse Fourier transform.

Step5: The corresponding phase profiles from the previous iterations are added to generate a multi-plane focal spot generation.

Step6: The iterative procedure continues until a specific iteration (n) value is obtained.

The loop iteration value (n) chosen here is 40; for more details about choosing the n value and the GS algorithm, please refer^1- 3^.

1. **Experimental setup**

The IDT imaging system comprises two systems as shown in Fig. S2; the green beam represents system1, which is the holographic optical tweezers system, and the red beam represents system2, which is the imaging system.

System1, uses a diode-pumped solid state (DPSS) laser emitting at 532 nm (Coherent, Verdi-V5) as the source. The source beam is spatially filtered, collimated, and incident on a reflective-type phase-only spatial light modulator (SLM) (Jasper display Corp, pixel number: 1920×1024; pixel pitch: 6.4 µm). A 4F system is constructed after SLM and an objective lens (Olympus, NA = 1.4, oil immersion) is used to focus the trapping beam on the sample^4^. The optical power of the trapping beam was less than 10 mW, which even cannot raise to 0.5^o^C on the free-floating medium, and this is far below the threshold level to damage the cell^5^. System2, the imaging system, comprises a Mach-Zehnder interferometer with off-axis configuration.


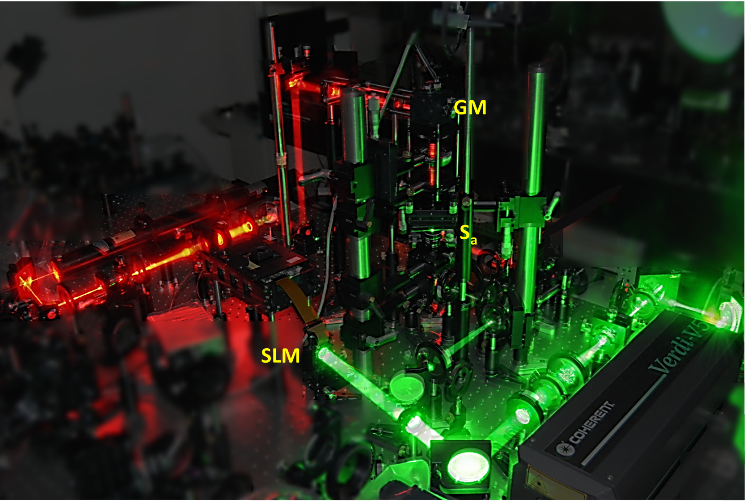


Fig. S2. IDT experimental setup: SLM: spatial light modulator GM: Galvo mirror, S_a_: sample.

A He-Ne laser emitting at 632.8 nm is spatially filtered, collimated, and split into a probe beam and reference beam by the beam splitter. The probe beam illumination is controlled by a galvo-mirror, and a condenser lens (Olympus, NA = 1.4, oil immersion) is used to control the illumination beam incident on the sample.

The diffracted wavefronts are collected by the objective lens (Olympus, NA = 1.4, oil immersion) and recombined with the reference beam; the holograms are recorded by the complementary metal-oxide-semiconductor, CMOS camera (1280 × 1024 pixel; pixel size: 5.2 μm). The different views of IDT imaging system are shown in Fig. S3.

The schematic of the optical tweezers system is shown in Fig. S4. The designed CGHs are loaded into the phase-only SLM and a telescope system is embedded after the SLM. The output is tightly focused by an objective lens to create the trapping spot on the sample at the trapping plane.

The generated trapping spots P_1_ and P_2_ at the trapping plane corresponding to x and y-axis are captured and shown in Fig. S5, where P_1_ and P_2_ is the fixed trap and rotation trap respectively. We arrest the live cell movements by the trapping beams. But still the cell has slight movement which is negligible and can be easily compensated by simple numerical algorithm during the reconstruction process and for the three dimensional image (3D) reconstruction procedures, refer to our earlier work^4^.


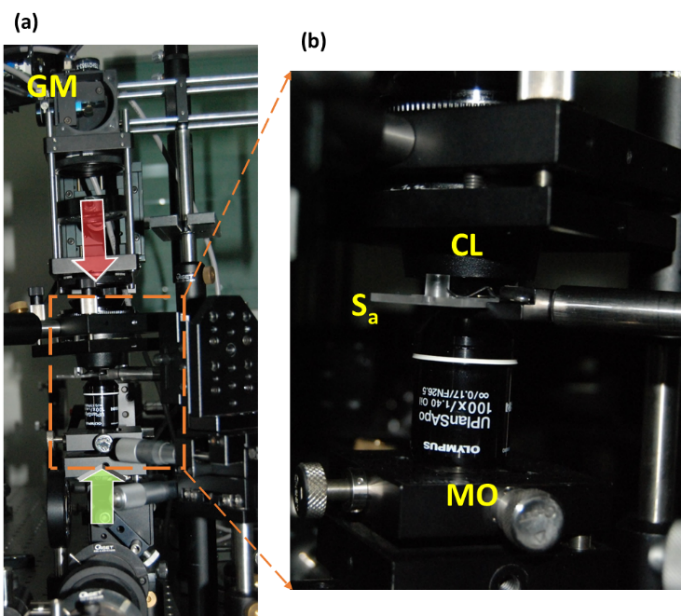


Fig. S3. Different views of IDT experimental setup. (a) View of probe beam arm, red and green arrow represents the probe and tweezer beam propagation directions towards the sample. (b) Zoomed view of dotted representation in (a) shows the sample arrangement in between condenser lens and the objective lens. GM: galvo mirror, CL: condenser lens, Sa: sample, MO: objective lens.


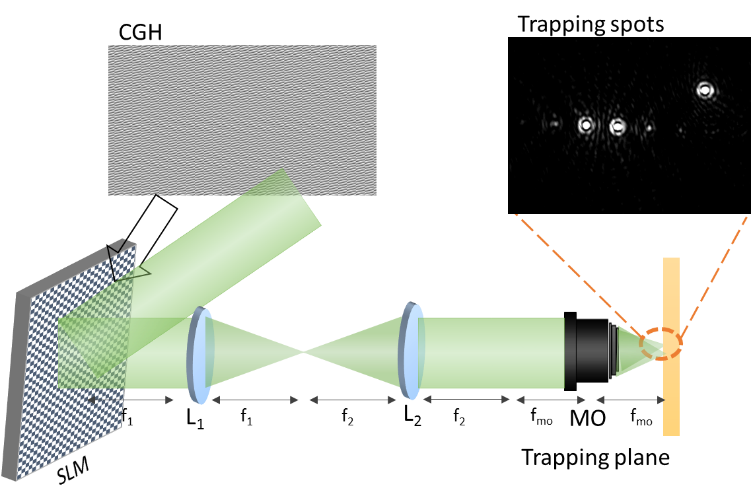


Fig. S4. Schematic of holographic optical tweezers system. MO: microscope objective.


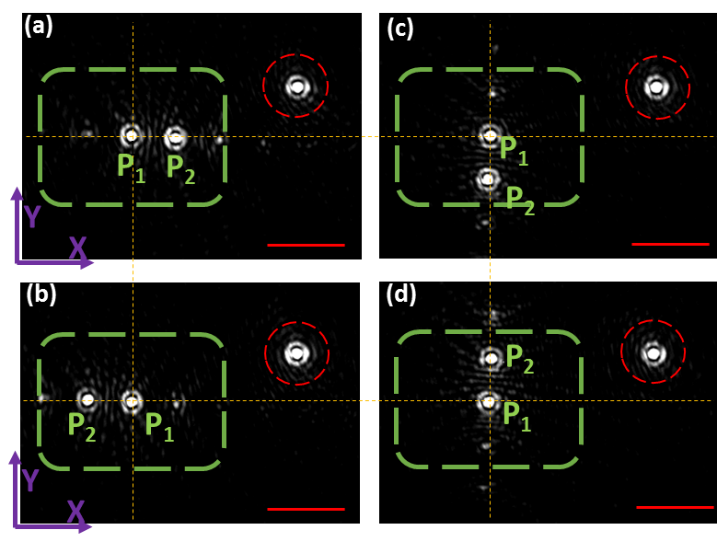


Fig. S5. Trapping spots shown at different rotation angles. (a) & (b) 90° and 270° rotation along x-axis, similarly (c) & (d) shows 90° and 270° rotation along y-axis, respectively. P_1_: fixed trap, P_2_: rotation trap, red circle: DC term. Scale bar: 10 µm. Visualization S1.

1. Estimation of the spatial resolution

To conduct the spatial resolution measurement from the reconstructed edge profile of the sample, this method is widely used to estimate the spatial resolution^4,7-10^. Therefore, we adopted this simple and less rigorous method for the resolution measurement. Fig S6. shows the lateral and axial fit obtained from the 3D reconstructed yeast sample. From the fitted profiles, the method gives lateral resolution of 150 nm and axial resolution of 310 nm. For more detailed explanation refer^7-9^.


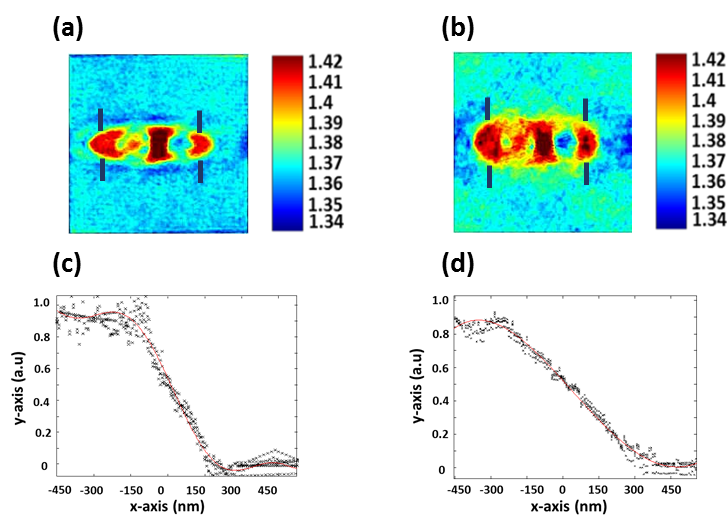


Fig S6. Sectional view of yeast and its edge response. (a) x-y sectional view; (b) y-z sectional view; (c) lateral fit marked on (a); (d) axial fit marked on (b).

References

1. Gerchberg, R. W. & Saxton, W. O. A practical algorithm for the determination of phase from image and diffraction plane pictures. Optik. **35**, 237-246 (1972).
2. Whyte, G. & Courtial, J. Experimental demonstration of holographic three-dimensional light shaping using Gerchberg-Saxton algorithm. New J. Phys. **7**, 117 (2005).
3. Gaunt, A. L. &. Hadzibabic, Z. Robust digital holography for ultracold atom trapping. Sci. Rep. **2**, 721 (2012).
4. Lin, Y. C., Chen, H. C., Tu, H. Y., Liu, C. Y., & Cheng, C. J. Optically driven full-angle sample rotation for tomographic imaging in digital holographic microscopy. Opt. Lett. **42**, 1321-1324 (2017).
5. Beauvoit, B. et al. Characterization of absorption and scattering properties for various yeast strains by time-resolved spectroscopy. Cell Biophys. **23**, 91-109 (1994).
6. Horstmeyer, R., Heintzmann, R., Popescu, G., Waller, L. & Yang, C. Standardizing the resolution claims for coherent microscopy, Nature Photonics **10**, 68-71 (2016).
7. M. Debailleul, V. Georges, B. Simon, R. Morin, and O. Haeberlé, High-resolution three-dimensional diffractive microscopy of transparent inorganic and biological samples. Opt. Lett. **34**, 79-81 (2009).
8. Choi, W. et al. Tomographic phase microscopy. Nature Methods. **4**, 717-719 (2007).
9. Bentzen, S. M. Evaluation of the spatial resolution of a CT scanner by direct analysis of the edge response function, Med. Phys. **10**, 579-581 (1983).
10. Simon, B. Tomographic diffractive microscopy with isotropic resolution. Optica. **4**, 460-463 (2017).
